# Ongoing Contact Activation in Patients with Hereditary Angioedema

**DOI:** 10.1371/journal.pone.0074043

**Published:** 2013-08-27

**Authors:** Joke Konings, Massimo Cugno, Chiara Suffritti, Hugo ten Cate, Marco Cicardi, José W. P. Govers-Riemslag

**Affiliations:** 1 Laboratory for Clinical Thrombosis and Haemostasis, Department of Biochemistry and Internal Medicine, Cardiovascular Research Institute Maastricht, Maastricht University Medical Centre, Maastricht, The Netherlands; 2 Internal Medicine, Department of Pathophysiology and Transplantation, University of Milan, IRCCS Fondazione Ca’ Granda, Ospedale Maggiore Policlinico, Milan, Italy; 3 Department of Biomedical and Clinical Sciences L. Sacco, University of Milan, Milan, Italy; University of Thessaly, Faculty of Medicine, Greece

## Abstract

Hereditary angioedema (HAE) is predominantly caused by a deficiency in C1 esterase inhibitor (C1INH) (HAE-C1INH). C1INH inhibits activated factor XII (FXIIa), activated factor XI (FXIa), and kallikrein. In HAE-C1INH patients the thrombotic risk is not increased even though activation of the contact system is poorly regulated. Therefore, we hypothesized that contact activation preferentially leads to kallikrein formation and less to activation of the coagulation cascade in HAE-C1INH patients. We measured the levels of C1INH in complex with activated contact factors in plasma samples of HAE-C1INH patients (N=30, 17 during remission and 13 during acute attack) and healthy controls (N=10). We did not detect differences in enzyme-inhibitor complexes between samples of controls, patients during remission and patients during an acute attack. Reconstitution with C1INH did not change this result. Next, we determined the potential to form enzyme-inhibitory complexes after complete *in vitro* activation of the plasma samples with a FXII trigger. In all samples, enzyme-C1INH levels increased after activation even in patients during an acute attack. However, the levels of FXIIa-C1INH, FXIa-C1INH and kallikrein-C1INH were at least 52% lower in samples taken during remission and 70% lower in samples taken during attack compared to samples from controls (p<0.05). Addition of C1INH after activation led to an increase in levels of FXIIa-C1INH and FXIa-C1INH (p<0.05), which were still lower than in controls (p<0.05), while the levels of kallikrein-C1INH did not change. These results are consistent with constitutive activation and attenuated depletion of the contact system and show that the ongoing activation of the contact system, which is present in HAE-C1INH patients both during remission and during acute attacks, is not associated with preferential generation of kallikrein over FXIa.

## Introduction

Hereditary angioedema (HAE) is a rare disorder predominantly caused by reduced levels or activity of C1 esterase inhibitor (C1INH) due to a mutation in the genes coding for C1INH (SERPING1). Patients with HAE experience episodic swellings that affect the subcutaneous and submucous tissues at the site of postcapillary venoles. Most common are asymmetrical cutaneous swelling of the hands, feet, face or genitals and swelling of the gastrointestinal tract. Swelling of the respiratory tract is less frequent, but potentially life-threatening [1]. Acute attacks of angioedema can be treated 1) by replacing C1INH with the plasma purified or recombinant protein; 2) by the plasma kallikrein inhibitor ecallantide or 3) by the specific antagonist of the bradykinin B2 receptor icatibant [1].

There are three types of HAE described: type I and type II are caused by either low levels of C1INH (type I) or dysfunctional C1INH (type II) (HAE-C1INH) [1]. Subjects with HAE type III have normal levels and activity of C1INH. In most of these patients the genetic cause of HAE is unknown (HAE-unknown). In one third, a point mutation (Thr328Lys or Thr328Arg) or a deletion (deletion of 72 base pairs: c.971_1018+24del72*) in the coagulation factor XII (FXII) gene is found (HAE-FXII) [2,3]. HAE-C1INH is predominantly and all HAE-FXII are inherited in an autosomal dominant fashion.

C1INH is a serine protease inhibitor and the main regulator of the classical complement pathway (named to complement C1) and the contact activation system [4]. The contact system, also known as the plasma kallikrein kinin system (PKKS), consists of FXII, prekallikrein and high molecular weight kininogen (HK). Activation of the contact system can initiate coagulation via activation of factor XI (FXI). C1INH is able to rapidly inhibit activated FXII (FXIIa), activated FXI (FXIa) and kallikrein [5,6]. It is the main endogenous inhibitor of FXIIa, kallikrein and FXIa: more than 90% of FXIIa, 50% of kallikrein and 50% of FXIa are inhibited by C1INH in plasma of healthy persons in *in vitro* experiments [6–8]. Other inhibitors of the contact system and FXIa are: α_1_-antitrypsin (AT) and α_2_-antiplasmin, which both inhibit FXIa for ~20-25% *in vitro* [8], and α_2_-macroglobulin (α_2_M). Approximately 35% of kallikrein is inhibited by α_2_M *in vitro*, however inhibition by C1INH is faster than inhibition by α_2_M [6,9].

The contact activation system is triggered *in vitro* when FXII is activated upon binding to negatively charged surfaces, such as dextran sulphate (DXS) or kaolin. Several physiological triggers of FXII have been identified, such as extracellular RNA and long-chain polyphosphates released from bacteria, however their contribution to activation *in vivo* is not yet clear [10,11]. Binding of the proteins of the contact system to endothelial cells initiates FXII-dependent conversion of prekallikrein into kallikrein [12]. FXIIa is able to activate both FXI and prekallikrein, HK is a nonenzymatic cofactor in these activations. Activation of FXI starts the intrinsic pathway of coagulation and results in the formation of thrombin and of a fibrin clot. Cleavage of prekallikrein by FXIIa generates kallikrein, which leads to the generation of bradykinin, via the cleavage of HK by kallikrein. Liberated bradykinin is the main mediator of symptoms in patients with HAE. Binding of bradykinin to the bradykinin B2 receptor on endothelial cells activates several intracellular signaling pathways that lead to vasodilatation, increased vascular permeability and fluid efflux [13,14].

During the attack phase of angioedema, activation of the contact system is observed: the levels of cleaved HK and FXIIa are elevated. The levels of prothrombin fragment 1.2 (a marker of thrombin generation) and D-dimer (a marker of fibrin degradation) are increased as well [15,16]. However, thrombotic complications during attacks or increased thrombotic risk in HAE-C1INH patients are not reported. It has been shown that activation of FXII by misfolded protein aggregates in patients with systemic amyloidosis leads to a form of FXIIa which activates prekallikrein but not FXI [17]. Hence, *in vivo*, activation of the kallikrein system without activation of the coagulation system can occur. This led to the hypothesis that in patients with HAE-C1INH, activation of FXII preferentially triggers prekallikrein activation, rather than FXIa generation by FXIIa.

To test our hypothesis, we measured activation of the contact system as 1) the levels of C1INH complexed with the activated contact factors in plasma samples, and 2) the *in vitro* potential of the plasma to form enzyme-inhibitory complexes when the contact system is completely activated. We used two different triggers of FXII, with different activation patterns, in separate samples. These measurements were performed in plasma obtained from HAE-C1INH patients during an attack and during remission and were compared with measurements in plasma from healthy controls.

## Materials and Methods

### Patients

In total, we analyzed 30 samples from patients with HAE-C1INH and 10 samples from healthy controls. These samples were obtained from 17 patients with HAE type I and 1 patient with HAE type II, and from 10 healthy volunteers. We examined 13 samples taken during an attack (obtained from 8 different patients) and 17 samples taken during remission from patients with HAE-C1INH. From 7 patients we analyzed both samples during attack and during remission.

Blood samples were collected in 3.2% sodium citrate as anticoagulant. Before blood drawing, the tourniquet was removed. The first 3 ml of blood was discarded. A subset of samples was also collected in an antiprotease mix to prevent activation of the contact system *in vitro*. The inhibitor cocktail was prepared by dissolving benzamidine (100 mM), hexadimethrine bromide (400 µg/ml), soybean trypsin inhibitor (STI) (2 mg/ml), leupeptin (263 µM) and aminoethylbenzenesulfonylfluoride (20 mM) in acid-citrate-dextrose (100 mM trisodium citrate, 67 mM citric acid, and 2% dextrose, pH 4.5). Blood was collected in a blood: anticoagulant/inhibitor cocktail ratio of 9:1 [18]. The samples were centrifuged at 2000g for 20 min at room temperature. Aliquots of the plasma samples were immediately snap-frozen in liquid nitrogen and stored at -80^o^C until use. Blood was collected in the course of routine diagnostic procedures and all patients gave oral informed consent that remaining plasma could be used for research purposes. If consent was given by the patient, the remaining plasma sample was labeled with a code which was documented into a data sheet. The Ethical Committee of the University of Milan approved of this study, also indicating that written informed consent is not necessary if the plasma is obtained during routine diagnostics and that this plasma can be used for research investigating the pathophysiology of hereditary angioedema.

### Materials

Dextran sulfate (DXS; Mr 500 000), ellagic acid, Hepes, STI and polybrene were from Sigma Chemical Co. (St Louis, Mo). The 96-well plates used were Nunc maxisorb (Denmark). Cetor^®^ was from Sanquin (Amsterdam, the Netherlands). The monoclonal antibodies were in house [19]. Normal platelet poor pooled plasma (University Hospital, Maastricht) consisted of plasma from 80 healthy volunteers.

The chromogenic assay for the measurement of C1INH activity was from Technoclone GmbH (Wien, Austria). Radial immunodiffusion assays for the detection of C1INH, C1q, and C4 antigen levels were from Siemens Healthcare Diagnostics (Munich, Germany). The chromogenic peptide kallikrein substrate S-2302 was from Chromogenix, Instrumentation Laboratory (Bedford, USA). For the measurement of cleaved HK the goat polyclonal anti-HK light chain antibody was from Nordic (Tilburg, the Netherlands) and the biotinylated rabbit anti-goat antibody and kallikrein from human plasma were from Sigma Aldrich Co. (St Louis, USA).

### Handling of the plasma

Inhibitory complexes were determined both directly in the plasma samples (basal samples) and in the plasma samples upon activation *in vitro* (activated samples). Furthermore, we reconstituted C1INH (cetor^®^) in the plasma samples.

To basal plasma samples we added either Hepes-buffer (25 mM Hepes, 150 mM NaCl, pH = 7.5) or C1INH, to inhibit free FXIa, FXIIa and kallikrein if present. The plasma samples were placed for 10 minutes at 37^o^C to allow optimal C1INH complex formation before stop buffer (containing STI and polybrene) was added. For activation of plasma samples we used either ellagic acid or DXS (Mr 500 000) for 15 min at 37^o^C. After activation of the plasma, we added C1INH or Hepes-buffer. To stop the reaction, we added stop buffer. Final concentrations of the above reagents were: 20% plasma, 0.2 U/ml C1INH, 0.1 mg/ml ellagic acid, 0.1 mg/ml DXS, 0.03% polybrene and 0.06 mg/ml STI. These samples were further diluted in the assay. Incubation of the plasma with ellagic acid or DXS leads to the activation of FXII. FXII activation with ellagic acid leads to activation of prekallikrein and FXI, whereas FXII activation with DXS only leads to activation of prekallikrein and not of FXI.

### Laboratory measurements

The assays for the detection of amidolytic activity of kallikrein in plasma and capacity of plasma to inhibit kallikrein amidolytic activity are based on a method described by Gallimore et al [20] using the chromogenic substrate S-2302. The presence of kallikrein amidolytic activity was measured by diluting the plasma 1:20 in 0.05 M Tris, 0.11 M NaCl (pH = 7.8), incubating it with the chromogenic substrate for 30 minutes at 37^o^C and the rate of release of paranitroaniline (pNA) was measured photometrically at 405 nm. To determine the capacity of plasma to inhibit the amidolytic activity of exogenous kallikrein, a fixed amount (0.06 U/ml) of purified human plasma kallikrein was added to serial dilution of plasma. The release of pNA was recorded after 5 minutes incubation at 37^o^C with the substrate. The capacity of plasma to inhibit amidolytic activity of kallikrein was expressed as percentage of normal using a standard curve of normal human pooled plasma.

Cleaved HK was evaluated with sodium dodecylsulfate-polyacrylamide gel electrophoresis (SDS PAGE) and immunoblotting analysis, as described previously [21].

### Enzyme-linked immunosorbent assay (ELISA)

The levels of C1INH in complex with FXIIa, kallikrein and FXIa (FXIIa-C1INH, KAL-C1INH, FXIa-C1INH) and AT in complex with FXIa (FXIa-AT) were determined by ELISA as described previously [19]. In short, the monoclonal antibody (mAB) KOK-12 was used as a capture antibody for C1INH complexed with FXIIa or kallikrein. FXIIa-C1INH was recognized with the mAB F3 and KAL-C1INH with the mAB K15. The mAB XI-5 was used as a capture mAB for FXI(a), the mAB R11 recognized FXIa-C1INH and the mAB AT-15 recognized FXIa-AT. Absorbance was read at 450 nm on an EL 808 Ultra microplate reader (Bio-tek Instruments Inc., Winooski, Vt).

Results are expressed in arbitrary units (A.U.) relative to a standard curve obtained from normal pooled plasma maximally activated with DXS (for FXIIa-C1INH and KAL-C1INH), or activated with ellagic acid (for all four complexes: FXIIa-C1INH and KAL-C1INH, FXIa-C1INH and FXIa-AT).

### Statistical analysis

The data are expressed as median [interquartile range (IQR)]. Differences between three or more groups were determined using the Kruskal-Wallis test with Dunn’s post hoc test. To determine if addition of C1INH to samples had an effect on the enzyme-inhibitory complex levels, the Wilcoxon Signed Rank Test was used. Correlations are expressed as Spearman’s coefficient. Results were viewed to be statistically significant different when p < 0.05. Statistical analyses were performed using IBM SPSS Statistics 20 for Windows (Armonk, New York: IBM Corp.) and Prism for Windows 5.00 (GraphPad Software Inc., San Diego, CA, USA).

## Results

The activity of C1INH, which is the main inhibitor of FXII activation and FXIIa, is low in plasma samples of HAE-C1INH patients, representing a risk for contact activation during blood drawing and storage. Therefore, we determined if the storage and preservation of plasma samples from patients with HAE-C1INH led to activation of the contact system. We compared samples taken at the same time in citrate alone and in an inhibitor cocktail mixture which efficiently prevents *in vitro* activation of the contact system [18]. We measured the levels of FXIIa-C1INH, kallikrein-C1INH and FXIa-C1INH in plasma samples obtained from 10 patients and from 10 healthy individuals. Comparison of these levels showed no difference between the two anticoagulants, both for the healthy individuals and for the HAE-C1INH patients (see Table 1). Therefore, low levels of C1INH in citrated plasma alone did not lead to contact activation after blood drawing and we were able to use plasma samples obtained from citrated blood for our study.

**Table 1:** Comparison of the levels of enzyme-inhibitory complexes in samples taken in citrate and in inhibitor cocktail as anticoagulant.

		FXIIa-C1INH (A.U.)	Kallikrein-C1INH (A.U.)	FXIa-C1INH (A.U.)
		Median [IQR]	Median [IQR]	Median [IQR]
HAE-C1INH patients	Citrate	0.73 [0.62-0.79]	0.5 [0.5 - 0.5]	1.37 [1.24-1.42]
	Inhibitor cocktail	0.75 [0.67-0.79]	0.5 [0.5 - 0.5]	1.28 [1.17-1.44]
Healthy controls	Citrate	0.62 [0.5-0.66]	0.5 [0.5 - 0.5]	1.31 [1.20-1.47]
	Inhibitor cocktail	0.64 [0.58-0.68]	0.5 [0.5 - 0.5]	1.26 [1.14-1.34]

C1INH: C1 esterase inhibitor; HAE: hereditary angioedema, IQR: interquartile range; A.U: arbitrary units

### Study population

Table 2 summarizes the main laboratory indices of the study population. The sex distribution is comparable between the groups, however the healthy controls are younger than the patients with HAE-C1INH. The C1INH antigen and activity levels were reduced in patients and consequently, cleaved HK and the spontaneous kallikrein activity were increased and the capacity to the plasma to inhibit kallikrein decreased compared to healthy controls.

**Table 2:** General characteristics of the study population.

	Healthy controls	HAE-C1INH (remission)	HAE-C1INH (acute attack)
	Median [IQR]	Median [IQR]	Median [IQR]
N	10	17	13
Gender (female)	9	13	10
Age (years)	21 [20-21]	48 [45-70]	48 [47-63]
C1INH function (%)	75.5 [69.8-82.5]	11.0 [0.0-18.5]	0.0 [0.0-5.75]
C1INH antigen (%)	100 [90-109]	25.0 [25.0 - 25.0]	9.0 [0.0-25.0]
Cleaved HK (%)	33.2 [30.4-38.0]	47.7 [42.2-48.1]	61.8 [56.5-70.3]
Capacity of the plasma to inhibit kallikrein (%)	120.8 [111.8-126.5]	56.0 [18.6-57.5]	25.5 [1.31-34.5]
Spontaneous kallikrein activity (mU/ml)	0.73 [0.44-1.89]	6.08 [2.15-13.0]	4.34 [2.09-16.21]
FXIIa-C1INH (A.U.)	0.62 [0.5-0.65]	0.72 [0.62-0.83]	0.67 [0.51-0.78]
Kallikrein-C1INH (A.U.)	0.5 [0.5-0.51]	0.5 [0.5 - 0.5]	0.5 [0.5-1.30]
FXIa-C1INH (A.U.)	1.31 [1.20-1.47]	1.40 [1.29-1.47]	1.33 [1.27-1.44]
FXIa-AT (A.U.)	0.66 [0.5-0.74]	0.83 [0.68-0.87]	0.84 [0.75-0.97]

C1INH: C1 esterase inhibitor; HAE: hereditary angioedema, IQR: interquartile range; HK: high molecular weight kininogen; A.U: arbitrary units

We did not observe differences in the levels of the enzyme-inhibitory complexes between healthy controls and patients, both in samples taken during remission and in samples taken during an acute attack of angioedema (see Table 2). Furthermore, we substituted C1INH in these plasma samples, and allowed free FXIa, FXIIa and kallikrein in these samples to form complexes with C1INH. This had no effect on the complex levels determined (data not shown).

### Activation of samples from patients

**Figure 1:**
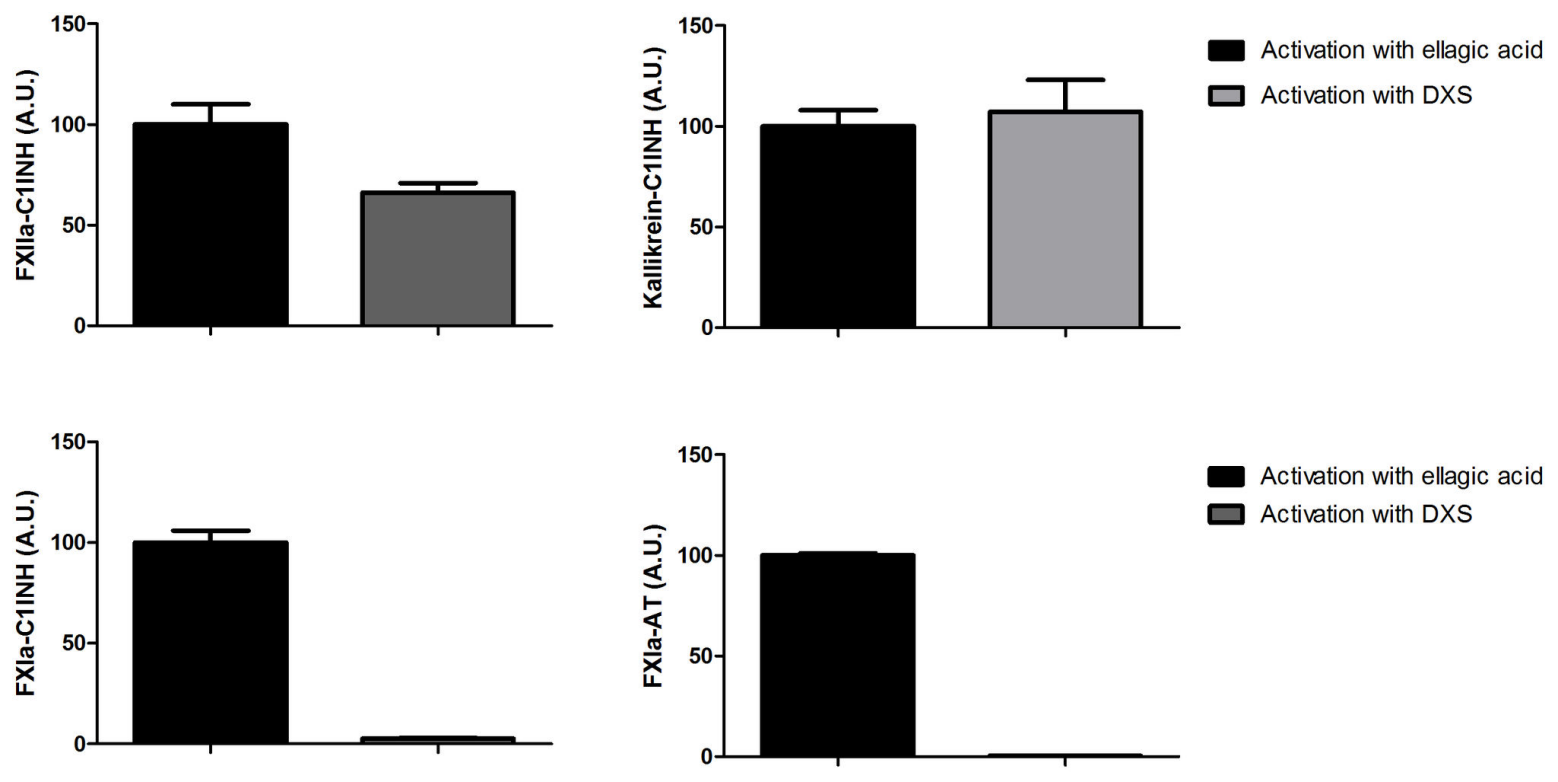
Enzyme-inhibitory complexes in normal pool plasma after activation with ellagic acid or dextran sulphate. The levels of inhibitory complexes were measured by ELISA after activation of normal pool plasma with either ellagic acid or dextran sulphate (DXS; Mr 500 000). Normal pool plasma was incubated for 25 minutes with either ellagic acid (0.25 mg/ml) or DXS (0.25 mg/ml) at 37^o^C in Hepes-buffer (25 mM Hepes, 150 mM NaCl, pH = 7.5). The reaction was stopped with stop buffer containing STI and polybrene. The volume of plasma was 50% of total volume during the activation with ellagic acid or DXS, and diluted further in the assay. A.U. = Arbitrary units.

Since we did not observe a difference between the levels of the activated contact factors in complex with their inhibitors in the basal samples, we wanted to determine the potential to form enzyme-inhibitory complexes after complete activation of FXII. We used two different activators of FXII, namely ellagic acid and DXS. Ellagic acid is a potent activator of FXII and is commonly used as a reagent in the activated Partial Thromboplastin Time (aPTT) test. DXS is mainly used in research as a FXII activator [22]. As shown in Figure 1, in normal pool plasma activation of FXII with ellagic acid activates both prekallikrein and FXI, whereas FXII activated with DXS only activates prekallikrein and not FXI. Since patients with HAE-C1INH have low levels of functional C1INH we measured the enzyme-inhibitor complexes directly after activation and after addition of C1INH (1 U/ml plasma) to the activated plasma in order to inhibit remaining, free FXIIa, kallikrein and FXIa.

#### Activation with ellagic acid

**Figure 2:**
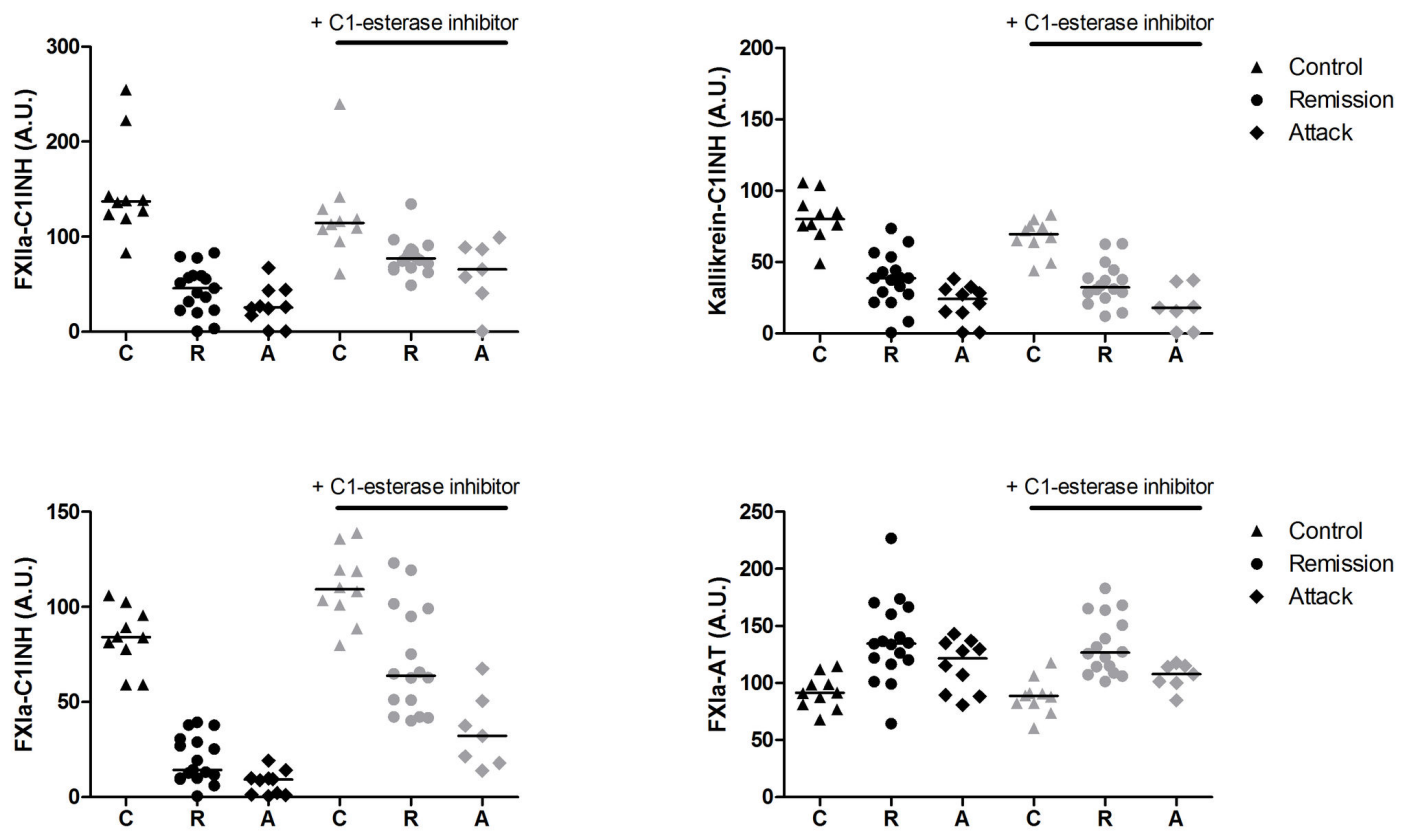
Enzyme-inhibitory complexes after activation with ellagic acid in samples from patients and healthy controls. Levels of enzyme-inhibitory complexes were measured by ELISA in samples from patients with HAE-C1INH and healthy controls after activation with ellagic acid, without (black) and with (grey) addition of C1 esterase inhibitor (C1INH) after activation. Plasma was activated with ellagic acid (0.25 mg/ml) for 15 minutes, than C1INH or Hepes-buffer (25 mM Hepes, 150 mM NaCl, pH = 7.5) was added and after 10 minutes stop buffer containing STI and polybrene was added. The volume of plasma was 50% of total volume during the activation with ellagic acid, and diluted further in the assay. Significant differences are indicated in Table 3. The line represents the median value; A.U. = Arbitrary units.

Figure 2 and Table 3 show the levels of the inhibitory complexes in patients with HAE-C1INH and in healthy controls after activation with ellagic acid. The levels of enzyme-C1INH complexes were higher in plasma samples from healthy individuals than in the plasma samples of patients, both in those taken during remission and those taken during an acute attack. The levels of FXIIa-C1INH and FXIa-C1INH increased significantly after addition of C1INH, indicating that not all FXIIa and FXIa were inhibited after activation with ellagic acid if no additional C1INH was added. However, the levels of the inhibitory complexes remained lower than those in healthy controls. The levels of kallikrein-C1INH did not increase if C1INH was added after activation. In comparison with healthy controls, the levels of FXIa-AT after activation with EA were higher in patients during remission and as expected these levels did not change after addition of C1INH.

**Table 3:** Enzyme-inhibitory complexes in HAE-C1INH patients and healthy controls after activation with ellagic acid.

	FXIIa-C1INH (A.U.)	Kallikrein-C1INH (A.U.)	FXIa-C1INH (A.U.)	FXIa-AT (A.U.)
	median [IQR]	median [IQR]	median [IQR]	median [IQR]
**No addition of C1-esterase inhibitor**				
Healthy controls (n = 10)	137.2 [122.6-162.7]	80.0 [74.1-93.1]	84.1 [73.1-97.3]	91.3 [80.3-102.2]
HAE-C1INH remission (n = 17)	45.9 [22.8-59.0]*	38.7 [24.6-48.9]*	14.3 [9.9-29.8]*	134.5 [118.4-163.5]*
HAE-C1INH attack (n = 10)	25.5 [13.3-43.6]*	24.1 [11.1-31.3]*	9.2 1.30-11.0]*	121.6 [89.1-135.6]
**Addition of C1-esterase inhibitor**				
Healthy controls (n = 10)	114.7 [104.6-132.4]	69.7 [60.3-76.3]	109.2 [97.9-123.5]	88.6 [80.0-95.0]
HAE-C1INH remission (n = 16)	77.10 [67.6-86.6]* #	32.3 [25.7-42.9]*	63.8 [44.4-98.1]* #	126.6 [110.3-160.6]*
HAE-C1INH attack (n = 7)	65.7 [40.7-89.0]* #	17.9 [0.6-36.5]*	32.2 [17.9-50.5]* #	107.8 [100.0-115.1]

C1INH: C1 esterase inhibitor; HAE: hereditary angioedema, IQR: interquartile range; A.U: arbitrary units

* Significant increase or decrease in level of inhibitory complexes compared to healthy controls (p < 0.05)

# Significant increase in inhibitory complexes compared to no addition of C1 esterase inhibitor (p < 0.05)

Since C1INH is an important inhibitor of kallikrein *in vivo*, we examined if the capacity of the plasma to inhibit kallikrein and the spontaneous kallikrein activity of the plasma correlate with the levels of kallikrein-C1INH complexes. In activated plasma samples, we observed a positive correlation between the levels of kallikrein-C1INH and the capacity of the plasma to inhibit kallikrein (r = 0.85, p < 0.001), and a negative correlation with the spontaneous kallikrein activity (r = -0.66, p = 0.001).

#### Activation with dextran sulphate

**Figure 3:**
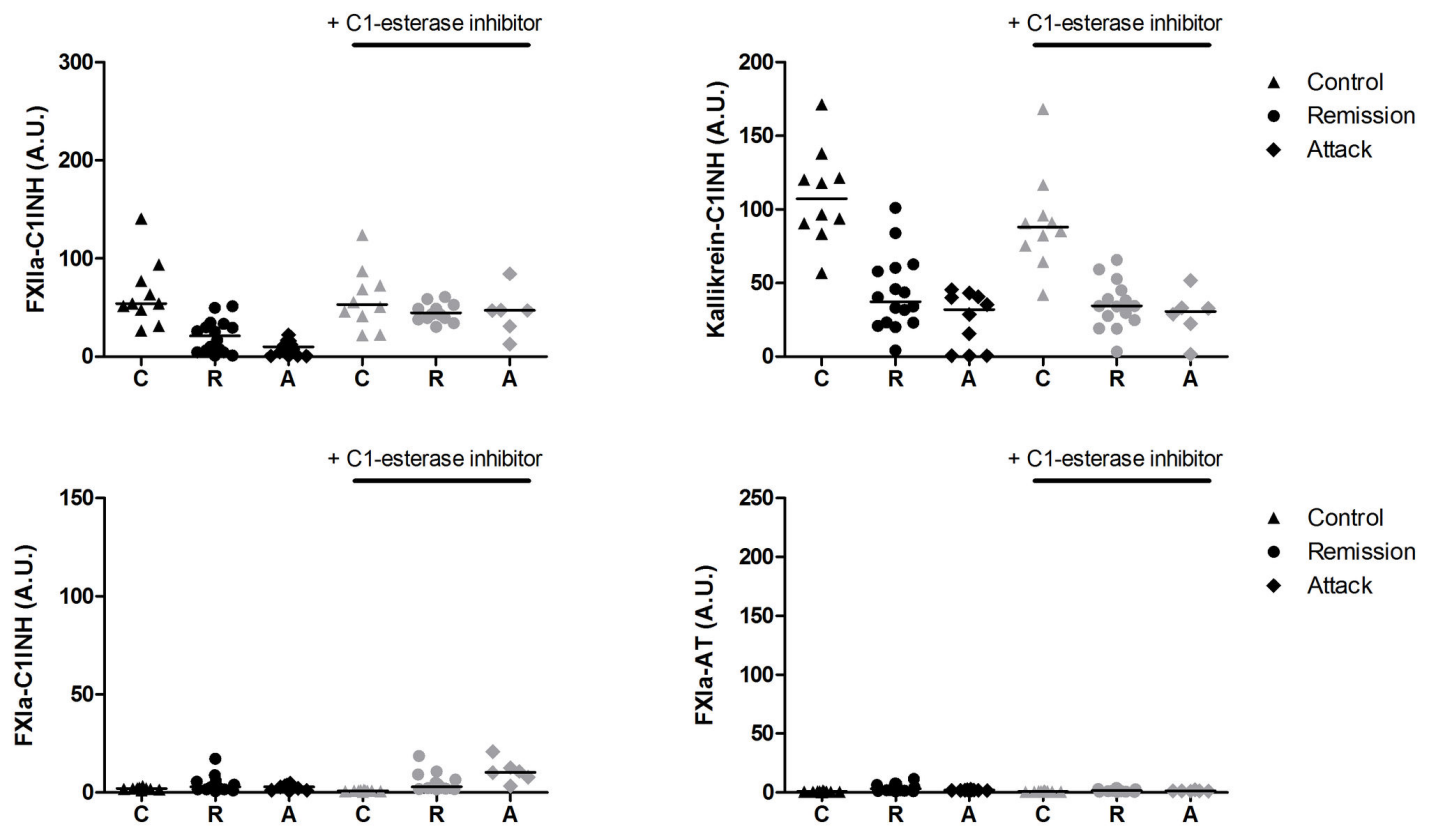
Enzyme-inhibitory complexes after activation with dextran sulphate in samples from patients and healthy controls. Levels of enzyme-inhibitory complexes were measured by ELISA in samples from patients with HAE-C1INH and healthy controls after activation with dextran sulphate (DXS; Mr 500 000), without (black) and with (grey) addition of C1 esterase inhibitor (C1INH) after activation. Plasma was activated with DXS (0.25 mg/ml) for 15 minutes, than C1INH or Hepes-buffer (25 mM Hepes, 150 mM NaCl, pH = 7.5) was added and after 10 minutes stop buffer containing STI and polybrene was added. The volume of plasma was 50% of total volume during the activation with DXS, and diluted further in the assay. Significant differences are indicated in Table 4. The line represents the median value; A.U. = Arbitrary units.

Next, we activated the plasma samples with DXS (see Figure 3 and Table 4). As expected, activation of plasma with DXS led to low levels of FXIa-C1INH and FXIa-AT compared to activation with ellagic acid. The levels of FXIa-C1INH were comparable between healthy controls and HAE-C1INH patients. The levels of FXIa-AT were higher in HAE-C1INH patients compared to healthy controls, however for both groups the median levels were low (less than 4 A.U. compared to approximately 100 A.U. after activation with ellagic acid). The results for kallikrein-C1INH after activation with DXS were largely comparable to those after activation with ellagic acid. The levels of kallikrein-C1INH were highest in healthy controls, there was no difference in these levels between remission and attack samples. The level of kallikrein-C1INH complexes did not increase after addition of C1INH after activation of the plasma. The levels of FXIIa-C1INH were highest in healthy controls and this was significant if no C1INH was added after activation. Addition of C1INH led to higher levels of FXIIa-C1INH in HAE-C1INH patients, due to this there was no difference in FXIIa-C1INH levels between healthy controls and HAE-C1INH patients. 

**Table 4:** Enzyme-inhibitory complexes in HAE-C1INH patients and healthy controls after activation with dextran sulphate (Mr 500 000).

	FXIIa-C1INH (A.U.)	Kallikrein-C1INH (A.U.)	FXIa-C1INH (A.U.)	FXIa-AT (A.U.)
	median [IQR]	median [IQR]	median [IQR]	median [IQR]
**No addition of C1-esterase inhibitor**				
Healthy controls (n = 10)	54.1 [43.7-81.4]	107 [88.7-125.6]	2.00 [1.62-2.30]	0.65 [0.5-0.96]
HAE-C1INH remission (n = 16)	21.1 [4.91-32.4]*	37.3 [23.1-59.8]*	2.86 [1.60-5.19]	3.19 [1.64-5.92]*
HAE-C1INH attack (n = 10)	8.15 [0.5-13.9]*	31.9 [0.5-41.2]*	2.83 [1.06-3.69]	1.90 [1.66-2.01]*
**Addition of C1-esterase inhibitor**				
Healthy controls (n = 10)	53.0 [36.6-76.1]	88.0 [72.7-101.1]	0.85 [0.69-1.05]	0.73 [0.54-0.99]
HAE-C1INH remission (n = 15)	44.7 [39.5-52.8] #	34.4 [24.8-45.1]*	2.78 [1.95-6.50]*	1.66 [0.72-2.53]*
HAE-C1INH attack (n = 6)	47.0 [26.3-56.7] #	30.8 [17.3-37.8]*	10.4 [6.61-14.6]* #	1.31 [1.09-1.60]

C1INH: C1 esterase inhibitor; HAE: hereditary angioedema, IQR: interquartile range; A.U: arbitrary units

* Significant increase or decrease in level of inhibitory complexes compared to healthy controls (p < 0.05)

# Significant increase in inhibitory complexes compared to no addition of C1 esterase inhibitor (p < 0.05)

## Discussion

The control of the contact system is defective in patients with HAE-C1INH due to a deficiency in C1INH. Activation of the contact system triggers the kinin pathway and, as a consequence, bradykinin formation is poorly controlled. Contact activation may also lead to activation of the intrinsic pathway of coagulation, however, HAE-C1INH patients have no increased tendency to thrombosis. One mechanism may be a preferential generation of bradykinin and less activation of the intrinsic coagulation route, but such mechanisms have not been explored. Therefore, we investigated if contact activation in these patients mainly leads to prekallikrein activation and less to FXI activation.

We determined the activation of the contact system in HAE-C1INH plasma samples by measuring the levels of C1INH complexed with FXIIa, kallikrein and FXIa. In accordance with previous findings from Cugno et al. [23], we did not observe a difference in the basal levels of the inhibitory complexes in healthy controls compared to samples of patients with HAE-C1INH during remission. In a study in five Norwegian patients with HAE-C1INH, it was found that the levels of FXIa-C1INH and kallikrein-C1INH were comparable during remission and during an attack of angioedema [24]. Our results agree with these findings and furthermore we observed that FXIIa-C1INH was not increased during an attack of angioedema in these patients. Since these measurements were performed in plasma with low levels of C1INH, possibly limiting complex formation, we also added C1INH to the samples before measurement of the enzyme-inhibitory complexes. We have shown that exogenous C1INH is able to form complexes, however, the addition of C1INH had no effect on the levels of inhibitory complexes in the basal samples. This indicates that no uninhibited activated contact factors were present in the samples. Other pathways of bradykinin formation exist, however, contact system activation is the main mediator of attacks of angioedema according to literature [25]. Possibly, FXIIa was formed but we were unable to measure it because C1INH cannot (efficiently) inhibit FXIIa, when it is bound to endothelial cells [26]. Furthermore, enzyme-C1INH complexes are rapidly cleared from the circulation possibly obscuring differences in complex concentrations [27–29]. Kallikrein-α_2_-macroglobulin complexes, which are cleared at a slower rate than kallikrein-C1INH, were found to be increased in a patient with HAE-C1INH during three different acute attacks of angioedema by Kaufman et al [9].

Next, we activated FXII in the plasma samples with two FXII triggers: ellagic acid and dextran sulphate. Activation of FXII with DXS will only activate prekallikrein whereas activation of FXII with ellagic acid leads to activation of both prekallikrein and FXI. The rationale to use DXS was that an *in vivo* activator with the same properties might mimic the hereditary angioedema phenotype in which bradykinin formation appears to prevail over prothrombotic effects (at least without apparent increased risk of thrombosis). The levels of the inhibitory complexes after activation were highest in the control group for both triggers, as expected, but also increased substantially compared to baseline in the HAE-C1INH patients. This indicates that even during an acute attack not all plasma C1INH is consumed in HAE-C1INH, but this is not sufficiently capable to control local bradykinin formation. Activation of the plasma with ellagic acid led to substantial lower levels of enzyme-C1INH complexes in the plasma of patients with HAE-C1INH compared to healthy controls, and these levels remained lower after addition of C1INH (even though the levels of FXIIa-C1INH and FXIa-C1INH increased in patients after addition of C1INH). On the other hand, the levels of FXIa-AT were increased in HAE-C1INH patients compared to healthy controls. Activation of the plasma with DXS, led to larger differences in the levels of kallikrein-C1INH between patients and controls compared to activation of the plasma with ellagic acid. However, the difference in FXIIa-C1INH levels between patients and controls was smaller after activation with DXS compared to activation with ellagic acid, and was not statistically significant after addition of C1INH. Activation of the plasma with DXS, led to low levels of FXIa-inhibitor complexes. Possibly, two different types of FXIIa are formed with the different activators. α-FXIIa is able to activate both prekallikrein and FXI (as seen with activation with ellagic acid), whereas β-FXIIa only activates prekallikrein (as seen with activation with DXS). The lower levels of FXIIa, FXIa and kallikrein in complex with C1INH after activation of the plasma from HAE-C1INH patients, suggest a reduction in the levels of the zymogens of the contact system. Joseph et al showed that in plasma taken from HAE-C1INH patients during remission, FXII spontaneously activates [30]. FXIIa in turn activates prekallikrein and FXI, leading to consumption of these proteins. Since both kallikrein-C1INH and FXIa-C1INH were reduced in plasma from patients compared to plasma from controls, activation of FXII during attacks of angioedema in these patients did not predominantly lead to kallikrein formation. This is in contrast to patients with amyloidosis, where activation of FXII leads to the formation of kallikrein-C1INH, without activation of the coagulation system [17]. However, other inhibitors, such as α_2_-macroglobulin for kallikrein and α_1_-antitrypsin for FXIa are also present in the plasma. The inhibition of kallikrein by α_2_M is slower than inhibition by C1INH, which could explain why the action of this inhibitor is not sufficient in HAE-C1INH patients to prevent attacks [9]. However, we activated the plasma during 15 min with ellagic acid or DXS, allowing kallikrein-α_2_M complexes to form. Only after these 15 min, C1INH was added to the sample. Therefore, we probably did not see a difference in kallikrein-C1INH levels before and after addition of C1INH. The fact that activation of FXI in these patients does not lead to an increase in thrombosis, may be due to the action of α_1_-antitrypsin (first described by Heck et al [31]) and other inhibitors of the coagulation enzymes downstream in the cascade. C1INH inhibits the two key enzymes of the kinin system efficiently: approximately 90% of FXIIa and 50% of kallikrein are inhibited by C1INH *in vitro*, while on the coagulation side it provides approximately 50% inhibition of FXIa [6–9],, but several other proteins control the cascade. The levels of FXIa-AT after activation were higher in patients than in healthy controls, highlighting the action of this inhibitor. Thus, even if we could not demonstrate a preferential activation of the kinin system, our data are consistent with the fact that the coagulation pathway is adequately controlled in patients with HAE-C1INH. Furthermore, increased fibrinolysis could protect HAE-C1INH patients during an acute attack against thrombosis. Increased levels of plasmin α2-antiplasmin (PAP) complexes have consistently been observed in patients during an acute attack of angioedema [24,32,33].

FXIIa independent pathways of formation of bradykinin from HK have been demonstrated. Prolylcarboxypeptidase (PRCP) [34] and Heat Shock Protein-90 (HSP90) [35] can directly activate prekallikrein, in complex with HK, bound to endothelial cells, independent from FXII-activation and prekallikrein itself has been shown to acquire enzymatic activity when bound to HK sufficient to cleave HK [36]. Moreover, recent studies have shown that mannose-binding lectin-associated serine protease -1 (MASP-1) and to a lesser extent MASP-2, both generated after activation of the complement system, are able to digest HK even in the absence of kallikrein. Important to realize for HAE-C1INH patients is that both the activity of MASP-1 and MASP-2 are controlled by C1INH [37] and MASP-1 and MASP-2 levels are elevated during episodes of stress and infection, known initiators of angioedema episodes.

In conclusion, there was no difference in the basal levels of the enzyme-inhibitory complexes between remission and attack samples, indicating that change in contact activation investigated as C1INH protease complexes was not associated with the attack. We observed lower levels of the inhibitory complexes after complete *in vitro* activation of FXII in the plasma of HAE-C1INH patients compared to controls. These reduced levels point to lower levels of FXI, FXII and prekallikrein in these plasmas, probably caused by *in vivo* activation and consumption of these proteins. Since our measurements do not demonstrate preferential activation of the contact over the coagulation system in HAE-C1INH, the apparent absence of thrombotic complications during angioedema attacks is probably due to other regulatory mechanisms controlling the coagulation cascade (inhibitors of active coagulation factors) or increased fibrinolysis which compensates for the increase in coagulation. Consumption of C1INH in HAE-C1INH patients leads to an unstable equilibrium. Since C1INH controls several systems that influence bradykinin formation, any factor that contributes to an increase in bradykinin formation, such as activation of the contact system or MASP-1 or MASP-2, could cause an attack of angioedema. 
